# Exploring medication adherence in racial/ethnic minorities

**DOI:** 10.3389/fphar.2025.1670633

**Published:** 2025-12-18

**Authors:** Joronia Chery, Lisa Gualtieri, Ian Lee, Grace Sheng, Meera Singhal, Sebastian Z. Ramos

**Affiliations:** 1 Tufts University School of Medicine, Boston, MA, United States; 2 Cedars-Sinai, Los Angeles, CA, United States; 3 Harvard T H Chan School of Public Health, Boston, MA, United States; 4 Temple University Lewis Katz School of Medicine, Philadelphia, PA, United States; 5 Tufts Medical Center, Department of Obstetrics and Gynecology, Boston, MA, United States

**Keywords:** medication adherence, racial/ethnic minorities, qualitative research, home medication management, facilitators to adherence

## Abstract

**Introduction:** Medication adherence remains a persistent challenge, particularly among racial and ethnic minority populations who experience cultural, structural, and environmental barriers to consistent medication use. Although adherence has been widely examined, less is known about the everyday practices and experiences that shape medication-taking behaviors. This study sought to explore home medication management routines among adults from racial and ethnic minority groups, with an emphasis on perceived challenges and facilitators of adherence.

**Methods: **Semi-structured interviews were conducted with 20 adults from racial and ethnic minority backgrounds who were taking prescription medications. Interview transcripts were analyzed thematically to identify patterns in medication routines and factors influencing adherence.

**Results: **Participants described a range of behaviors that shaped day-to-day medication use. Facilitators included visual cues and anchoring medications to established daily routines, while disruptions in routine were a common source of missed doses. Nearly half of participants (45%) reported mentally retracing their steps later in the day to determine whether they had taken their medication. More than half (55%) described giving or receiving support from other household members. Family experiences and cultural beliefs, including mistrust of clinicians, reliance on herbal remedies, and a strong emphasis on self-care, emerged as additional influences on medication-related decisions.

**Discussion:** Findings illustrate how behavioral cues, household dynamics, and cultural beliefs shape medication adherence among racial and ethnic minority patients. Although limited by the small sample size, this study highlights opportunities for developing culturally responsive, behaviorally informed interventions that better integrate medication-taking into patients’ daily lives. Future research should build on these insights to inform scalable strategies that address persistent disparities in adherence.

## Introduction

Medication adherence remains a critical determinant of health outcomes, yet it is widely recognized as a persistent challenge across chronic conditions. The World Health Organization (WHO) defines adherence as “the degree to which the person’s behavior corresponds with the agreed recommendations from a healthcare provider” (Jimmy and Jose, 2011). High adherence has been associated with decreased morbidity and improved quality of life, whereas nonadherence contributes to approximately 50% of treatment failures, 125,000 deaths, and 25% of hospitalizations annually in the United States (Kim et al., 2018). Despite its clinical importance, adherence rates to chronic medications in developed countries are estimated at only 50% (Hincapie et al., 2019), underscoring the urgent need for strategies that address how patients manage medications in their everyday lives.

Current research on adherence often underrepresents patients from racial/ethnic minority populations (Hincapie et al., 2019). In the United States, these groups bear disproportionate burdens of chronic illness and complications. Studies show they are 1.5–2 times more likely than non-Hispanic White individuals to experience major chronic conditions (Price et al., 2013), and between 2004 and 2007, non-Hispanic Black patients experienced over 430,000 excess hospitalizations due to chronic health conditions compared to their White counterparts (Doshi et al., 2017). These disparities highlight the importance of understanding adherence in populations for whom cultural, linguistic, historical inequity and socioeconomic barriers intersect with systemic inequities in healthcare and medication access.

Adherence behaviors in racial/ethnic minority populations are shaped by complex and intersecting factors. Cultural norms, mistrust in health systems, limited access to pharmacies, and experiences of discrimination in care settings have been identified as barriers (Asiri et al., 2023; Bazargan et al., 2021; Rafanelli, 2021). At the same time, facilitators such as family support, community networks, and environmental cues—including habit stacking, placement of medicines within daily routines, and reminders—may promote adherence. These dynamics reinforce that adherence is not solely a matter of individual willpower, but rather emerges from the interaction of personal, cultural, and structural determinants.

Behavioral science frameworks provide further insight into these mechanisms. The Capability, Opportunity, Motivation–Behavior (COM-B) model emphasizes that adherence depends on patients’ skills and knowledge (capability), the availability of social and environmental supports (opportunity), and beliefs and intentions (motivation) (West and Michie, 2020). Similarly, the Health Belief Model (HBM) highlights the role of perceived risks, perceived benefits, barriers, cues to action, and self-efficacy in shaping adherence (Alyafei and Easton-Carr, 2025). Yet, few qualitative studies have applied these frameworks to minority populations, whose adherence is uniquely influenced by cultural practices, systemic barriers, and family support structures. Integrating behavioral science perspectives with the lived experiences of minority patients can deepen understanding of adherence and inform the design of culturally responsive interventions.

This study was therefore designed as a qualitative, exploratory investigation with a small, non-representative sample to generate insights rather than generalizable conclusions. Semi-structured interviews were conducted with adults from racial/ethnic minority backgrounds who were receiving chronic pharmacotherapy. Our aim was to explore how patients manage their medications at home across three critical stages of adherence: initiation, implementation, and persistence, the latter phase modified from “discontinuation” (Vrijens et al., 2012). Specifically, we sought to identify the challenges and facilitators encountered at each stage, and to examine how environmental cues, family support, and cultural contexts influence adherence behaviors. By foregrounding the lived experiences of minority patients and situating them within behavioral science frameworks, this study contributes to an evidence base that can guide the design of low-burden, culturally tailored strategies and inform future research directions.

## Methods

### Approach

Our goal for this cross-sectional study was to better understand how racial/ethnic minorities manage medication in their daily lives. We developed an interview guide and semi-structured interview process to explore their experiences with home medication management, including how they chose medication storage locations and established medication-taking habits. By asking participants to recount their medication routines, we aimed to identify factors that support or hinder adherence. Interview questions, which were derived from an earlier study with older adults (Gualtieri et al., 2022), were designed to address the three components of adherence‒initiation, implementation, and discontinuation, the latter of which we modified for the purposes of this study to persistence (Vrijens et al., 2012).

### Participants

Our study goal was to interview 20 people, deemed appropriate for an exploratory study. We recruited participants for the study via two modalities, flyers and ResearchMatch, both indicating the purpose of the study and that they would be compensated for their time with a $20 gift card. Study protocols were approved by Tufts University Health Sciences Institutional Review Board (IRB #00004335).

Onsite recruiting was conducted by posting study flyers at two Tufts University School of Medicine affiliated primary care facilities in multiple urban centers. Patients met inclusion criteria if they were 18 years of age and older, had no cognitive impairment, were prescribed at least one long-term oral medication, were English-proficient and identified as one of the following racial/ethnic minorities: Black, Hispanic/Latino, Asian-Pacific Islander, Native American. Participants taking exclusively non-oral medications, supplements and vitamins, short-term medication lasting less than 1 month, or medications administered exclusively by healthcare providers were excluded from the study.

**FIGURE 1:**
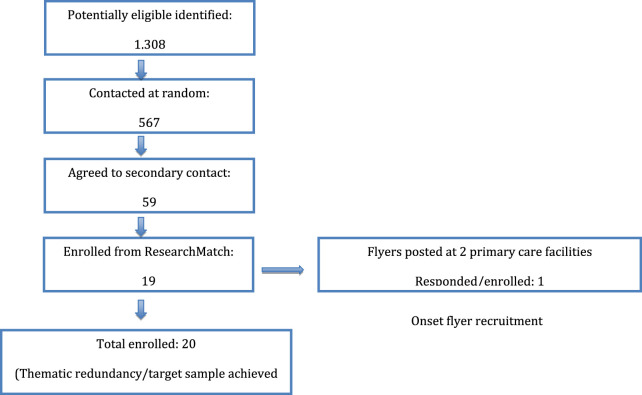
Recruitment flow diagram.

To recruit additional participants, we used ResearchMatch and entered several filter restrictions. For demographics, age was set to between age 18 and 110, and race/ethnicity was set to participants who identify as “American Indian or Alaska Native,” “Asian,” “Black or African American or African,” “Hispanic or Latino or Spanish,” “Middle Eastern or North African,” and “Native Hawaiian or other Pacific islander.” We recruited participants taking at least one long-term medication for chronic illness using the medication filter. Our search yielded 1308 eligible participants, of which 567 were contacted at random with an initial recruitment message. Of these, 59 agreed to secondary contact in the study consent form, and, of these, 19 enrolled. This brought the number of participants to 20 total at which point recruitment ceased. Recruitment is depicted in Figure 1.

### Interviews

Consent to participate in the study was obtained at recruitment, and consent to record the session was obtained at the start of each session. The interviews were scheduled for 30 min, although some lasted up to an hour. Sessions were conducted via Zoom videoconferencing with audio and video and only 1 participant was unable to use video.

JC conducted all interviews; JC received interview training from LG (PhD, experienced qualitative interviewer) that included role-playing, piloting the guide with two practice interviews, and iterative feedback. LG participated in interviews in a secondary role (asking brief clarifying questions only) and did not lead interviews. Neither JC nor LG had a prior therapeutic relationship with study participants. We maintained a reflexive journal during data collection and analysis to document interviewer impressions, potential sources of bias, and early analytic ideas; excerpts from these memos informed our coding meetings. We describe interviewer demographic characteristics (JC: female clinician-researcher; LG: female social-behavioral researcher) to allow readers to consider how interviewer positionality may have shaped responses.

Interviews were conducted over Zoom with password protection and recordings saved directly to the university’s secure, encrypted drive. Audio/video files were deidentified at the time of transcription; all personal identifiers were removed and substitute participant IDs assigned. Deidentified transcripts and analytic files were stored on encrypted institutional servers with access restricted to the study team. Participants were not asked to member-check transcripts or findings.

Recorded sessions were transcribed verbatim and all recordings and transcripts were deidentified and stored securely. Each interview began with the collection of simple demographic information such as age and race/ethnicity. Participants were next asked, “What role do you think race/ethnicity plays in your medication adherence?” Further prompting included, “What is your culture or family’s relationship with medicine?” Participants were then asked sets of questions covering the three components of adherence, specifically initiation (i.e., obtaining their medications), implementation (i.e., taking medications at home), and persistence (i.e., changes to routine or lifestyle and adherence to other domains). Participants’ self-reported medication adherence was calculated using the Morisky Medication Adherence 8 (MMAS-8) scale (Berlowitz et al., 2017; Bress et al., 2017).

### Analysis

Transcripts were coded using an inductive thematic approach (Clarke and Braun, 2013). JC and LG independently reviewed and coded transcripts using a living codebook in which codes were iteratively refined and definitions recorded. Disagreements were resolved in consensus meetings; where necessary, a third coder (MS) adjudicated. We did not calculate formal inter-rater reliability statistics (e.g., Cohen’s kappa) because the analysis followed an iterative, interpretive thematic process rather than a fixed checklist coding scheme; instead we focused on consensus coding and documented analytic decisions in memos. We maintained an audit trail consisting of (a) the living codebook with versioning, (b) analytic memos after each coding meeting, and (c) a log of decisions about code merges and theme selection. Excel was used to organize coded excerpts, frequencies, and exemplar quotations. Frequency counts are presented as descriptive indicators and are not used for inferential claims.

Responses to each question were extracted and grouped by archetypes. For example, if a participant reported storing medication in the kitchen for visibility, we categorized that under “Kitchen–Visual Cue.” Demographic data and MMAS-8 scores were used descriptively to contextualize themes, but no inferential statistical analyses were conducted due to the small sample size and qualitative nature of the study. As such, this study did not employ statistical hypothesis testing and no power calculation was performed—in line with best practices for exploratory qualitative research.

We summarized the frequencies of key responses related to medication storage, use of pill organizers, providing or receiving reminders, and strategies for remembering medication. Descriptive statistics were used solely to enhance the narrative understanding of themes. The themes, definitions, and quotes are in Table 1. The mapping of themes to COM-B and HBM constructs are in Table 2.

**TABLE 1:** Major themes, definitions, and exemplar quotes.

Theme	Definition	Frequency (N/20)	Exemplar quote
Visual cues / Visibility	Storing meds in places where they are easily seen to trigger taking them (counters, nightstands).	7/20	“I have to have them there where I can see things visually so I can look at them. that is one of my reminders.” — P#3
Routine linkage / Habit stacking	Pairing medication with established daily habits (skincare, meals, pet care).	5/20	“I take my medication around the time I do my nighttime skin routine.” — P#1
Mental replay / 'Did I take it?'	Mentally retracing day’s actions to determine if a dose was taken.	9/20	“At the end of the day, I just kind of review what I’ve done during the day and would realize I had not done that step.” — P#4
Change in routine as trigger for lapses	Missed doses when daily schedule is disrupted.	11/20	“Change in routine — that’s when I miss it...like an earlier appointment or travel.” — P#9
Co-adherence / family reminders	Providing or receiving help from household members to remember meds.	11/20	“I like to take my medicine at the same time my son takes his medicine. he’ll tell me ‘Dad go get your medicine right now.’” — P#10
Cultural/home remedies / mistrust	Family or cultural preference for herbal remedies or mistrust of clinicians shaping attitudes.	8/20	“We don’t like taking meds... my mom grew up on a farm.herbal remedies.” — P#19
Storage constraints / privacy concerns	Choosing storage based on need for privacy, available space, or concern about others.	6/20	“I keep them in the dresser because I don’t want my roommates to see them.” — P#17

Counts are descriptive and not used for inferential claims; exemplar quotes are representative excerpts from transcripts.)

**TABLE 2:** Mapping of themes to COM-B and HBM constructs.

Theme	COM-B construct(s)	HBM construct(s)	Rationale Linking Theme to construct	Exemplar quote
Visual cues / Visibility	Opportunity (physical)	Cues to action	Visible storage increases physical opportunity and provides external cue to action.	“I have to have them there where I can see things visually.” — P#3
Routine linkage / Habit stacking	Opportunity (physical), Capability (psychological)	Cues to action, Self-efficacy	Linking meds to established routines reduces cognitive load and creates predictable cues.	“I take it when I do my nighttime skin routine.” — P#1
Mental replay / 'Did I take it?'	Capability (psychological)	Cues to action	Cognitive strategy used to check past behavior; reliance on memory.	“I just kind of review what I’ve done during the day.” — P#4
Change in routine → lapses	Opportunity (physical/social)	Perceived barriers	Disruptions remove usual cues and increase barriers to adherence.	“When my routine changes I often forget.” — P#9
Co-adherence / family reminders	Opportunity (social), Motivation (reflective)	Cues to action; Perceived benefits	Social supports create accountability and shared benefits.	“My son will tell me ‘Dad go get your medicine right now.’” — P#10
Cultural/home remedies / mistrust	Motivation (reflective); Capability (knowledge)	Perceived susceptibility/benefits; Perceived barriers	Cultural beliefs and mistrust influence perceived benefits and barriers.	“My family relied on home remedies. we don’t like taking meds.” — P#19
Storage constraints / privacy	Opportunity (physical)	Perceived barriers	Limited space or privacy concerns limit feasible storage options.	“I keep them in the dresser for privacy.” — P#17

## Results

### Demographic data

We interviewed a total of 20 participants, of whom 75% were female and 25% were male. Regarding racial/ethnic minority identity, 75% of participants identified as Black or African American, 15% as Asian, and 10% as Hispanic or Latino. A plurality of participants (40%) were aged 50–59. The 40–49 and 70–79 age groups each accounted for 20%, followed by 15% in the 60–69 age range, and one participant (5%) in the 20–29 age range.

### Medication adherent behavior

Within the cohort, 85% of participants reported ever forgetting to take their medication, and 25% reported having recently forgotten to do so, defined as within the past 2 weeks according to MMAS-8. The average MMAS-8 adherence score was 4.88 and the median was 5 (range of 0–8).

### Medication storage location

Rooms used to store medication were bedrooms, bathrooms, kitchens, and workrooms or study spaces. Bedrooms were the most commonly reported, with 35% of participants noting placement within dresser drawers (10%), on top of dressers (5%), nightstands (5%), both dresser and nightstand (5%), and bedroom side tables (5%). One participant who used her dresser explained:

“[I] come home and when I am more settled around 9 o’clock I take my medication. My medication is stored on top of my dresser. I do my skin routine at bedtime and since my skin products and mirror are also on my dresser, I take my medication around the time I do my nighttime skin routine.” — Participant #1.

Bathrooms were the second most common location (30%), with participants storing their medications in places such as the vanity cabinet or drawer (10%), linen closet (5%), medicine cabinet (5%), vanity counter (5%), or on top of an ironing board (5%). Kitchens followed (20%), with storage on the top of the refrigerator (10%) and inside kitchen cabinets (10%). Two participants cited using a workroom or study space, one placing medication on a shelf and the other in a desk drawer.

Descriptive observations revealed variation in self-reported adherence across these locations. Among those who stored medications in the bathroom, 83% reported ever forgetting to take medication, and none reported recent lapses. In contrast, participants using the bedroom as a storage location reported 86% ever forgetting and 43% recently forgetting. All those using kitchen locations reported ever forgetting (100%), and 25% reported recent forgetting. The average adherence score was highest among participants who stored medications in the bathroom (6.58) and lowest for those using the bedroom (5.57).

### Medication storage containers

Of the cohort, 11 participants (55%) utilized a pill organizer. When comparing those who do not use pill organizers to those who do, 77% of the former group cited ever forgetting to take their medication while 91% of the latter group cited such. Similarly, 23% of the former group cited recently forgetting to take their medication and 18% of the latter group cited such. The average adherence score for pill organizer users was 6.02 compared to 5.36 for non-users, but no statistical significance was found on t-testing (p = 0.3626). Common rooms noted as storage locations for pill organizer users were the bedroom (45%), kitchen (36%), and bathroom (18%). The nightstand was the most popular location within the bedroom with 3 total participants, while the dresser and side table were each cited by one participant. Of the four participants who noted using the kitchen, all four cited placing their pill organizer on the kitchen counter. Two participants cited using the bathroom with one citing the bathroom shelf and the other citing the linen closet. Of those using pill organizers, medication adherence was highest in the bathroom group given an average adherence score of 7.5 followed by the bedroom with an average adherence score of 5.85 and was lowest in the kitchen counter group with an average adherence score of 5.5. No statistical significance was found when comparing the scores between groups.

#### Trends in decision making and routines

When asked about their reasons for selecting a medication storage location, participants described a variety of considerations. A frequently mentioned reason (n = 7) was the desire to place medications somewhere visually noticeable, such as the kitchen counter. One participant explained:

“I know that a lot of people like to store their medication in the medicine cabinet, but for me I think I would probably totally forget to be honest. I have to have them there where I can see things visually so I can look at them. I guess that is one of my reminders–being able to see things visually.” — Participant #3.

A participant who stored medication on their nightstand said:

“Because I can remember it if it’s right there in my face.” — Participant #17.

Five participants noted choosing locations tied to daily routines or specific activities. Examples included placing medications near skincare items for bedtime routines, on ironing boards for morning habits, or in locations associated with pet care. Other reasons for medication storage choices included general convenience, available space, pairing with meals or water, time spent in a particular location, privacy, and familial influence.

When discussing circumstances that led to forgetting to take medication, participants cited five themes: change in routine, stress or distraction, evening fatigue or falling asleep before taking medication, obstructed visibility of the medication, and simple forgetfulness.

Participants shared how they realized they had missed a dose with 45% describing mentally retracing their day. One participant shared:

“At the end of the day, I just kind of review what I’ve done during the day and would realize I had not done that step.” — Participant #4.

Other participants noted:

“Yes, I do not know if it’s just me, but I try to go through and remember ‘what did I do? Did I take it?’ .and I will try to remember the motion of taking the medicine. And so sometimes in the morning, I’ve done the motion of taking the 7 o’clock medicine but I’ll confuse that with oh you’ve already taken all of your medicine, so I’ll miss that second group of medicine.” — Participant #9.

“If I do not remember [to take my medication] then I will remember when I am eating my lunch. as a parent, I always go through my morning or my day [during lunch] and as I’m going through the day that’s when I remember I did not take my medicine.” — Participant #10.

### Providing or receiving adherence reminders

Participants were asked about their relationship with others in the context of medication management—specifically, whether they provided or received help with remembering to take medications. We term the providing or receiving medication reminders with family members “co-adherence.” Eleven participants (55%) indicated that they either helped someone else or received assistance themselves. Among the six participants who helped others, 78% reported ever forgetting to take their own medication, and 22% reported recently forgetting. Their average adherence score was 6.17. In comparison, participants who did not provide or receive assistance had a lower average adherence score of 5.72.

Two participants reported receiving assistance but not providing it. Their average adherence score was 4.13; both participants reported having ever forgotten, and one reported recently forgetting to take medication. Three participants reported both providing and receiving assistance. All three had ever forgotten, but none reported recently forgetting. Their average adherence score was 5.92.

Participants described various forms of support through co-adherence rituals. One participant reflected:

“I like to take my medicine at the same time my son takes his medicine–so it’s like a routine, he takes his I take mine. as a single dad I am always reviewing what needs to happen that day for my son and if I did not take my medicine that morning … I remember I did not take it with my son. and he’ll tell me ‘Dad go get your medicine right now.’” — Participant #10.

Another participant shared:

“I have one son that takes nighttime medication like me. He [my son] looks towards me to give him that particular [nighttime] medicine. there are times that if I’m busy he will stop me and say ‘Hey mom, it’s time for us to take our medicine’ and I’m like okay let me go ahead and stop what I’m doing, give him his medicine, and take mine.” — Participant #15.

These accounts illustrate how shared routines and reminders from family members can influence adherence behaviors in daily life.

### Roles of family, race/ethnicity, and culture

As noted above, in addition to collecting demographic information about race and ethnicity, participants were asked, “What role do you think race/ethnicity plays in your medication adherence?” and “What is your culture or family’s relationship with medicine?” These questions were designed to explore how familial and cultural factors may shape individuals' perspectives and behaviors related to medication use.

Participants identifying as Black/African American and Hispanic/Latino described familial reinforcement of herbal or home remedies over pharmaceuticals.

One participant explained:

“We do not like taking meds. my mom grew up on a farm, her dad was very knowledgeable in herbal remedies and my mom was as well and she taught us as well, and we do not like the fact that we’re not rich people and we did not always have the healthcare that we needed and it just seemed like modern times we just needed to go to the doctor for a lot of stuff that can be done for yourself that people do not explore it much anymore.” — Participant #19.

Another participant reflected on generational values regarding home remedies:

“My mom and dad really were not into modern medicine. I’m from a border town here in Texas. my family—both on my mom and dad’s side—really relied on home remedies. more so than prescription medicine. That’s what I observed all of my childhood. My values were home remedies—that’s what I learned, but as I got older, more educated, and moved away from my culture, it [my values] changed to prescription medication. With more research and experience I now find both are beneficial, not one more important than the other.” — Participant #10.

One Black/African American participant described a cultural emphasis on respect for medication-taking routines:

“My upbringing provided a healthy respect for it [taking medication]. there was some family reinforcement of traditional medicine and taking medication at a timely manner.” — Participant #4.

Others cited racialized experiences in the healthcare system as shaping their attitudes:

“I will not say I’m a big fan of going to doctors or hospitals, but I’ll go if I have to go. Unless I’m seriously ill, I’m not going to go. In my experience, I have not had the greatest feeling that doctors either have time or want to listen to what I have to say. and I think that’s mostly related to my race. there are little stories here and there [from family].” — Participant #8.

Some Black/African American and Hispanic/Latino participants noted that trust in Western medicine developed over time, often through counseling from healthcare providers or conducting personal research. However, families often emphasized self-care as a way to reduce reliance on medication.

One Asian participant described culturally embedded motivations for adherence:

“As an Asian, we are very in tune to our health, so when a doctor says you will need to take a medication, we will take it. Our desire is to live as long as we can so we can be involved with our grandchildren and great grandchildren–so medication is very important to us.” — Participant #5.

These responses reflect the diversity of cultural perspectives on medicine and illustrate how familial and cultural narratives may interact with individual adherence behaviors.

## Discussion

### Thematic insights across initiation, implementation, and persistence

Our findings illustrate that medication adherence unfolds across three interrelated stages: initiation, implementation, and persistence (Vrijens et al., 2012). During initiation, participants described challenges such as confusion about new prescriptions and competing life demands that delayed the start of treatment. In the implementation stage, adherence was shaped by how medications were incorporated into daily routines, with some participants linking medication-taking to routine household tasks or family reminders. Persistence with medication-taking, or lack of discontinuation, was supported by long-term trust in clinicians and internal motivation yet undermined by treatment fatigue and changes in life circumstances. Together, these thematic insights highlight that adherence is not a single decision, but rather an evolving process influenced by shifting personal and contextual factors.

### Cultural and social influences on adherence

Cultural and social influences were central to participants’ accounts of medication management. Several participants recalled family teachings that emphasized natural remedies or spiritual practices, which sometimes delayed or replaced biomedical treatment. Others described family members introducing pill organizers or serving as accountability partners, underscoring the importance of co-adherence and intergenerational support. These findings align with prior literature suggesting that cultural beliefs and social networks simultaneously serve as barriers and facilitators, shaping how adherence is experienced in everyday life.

### Cognitive and environmental cues in daily routines

Participants frequently described the use of cognitive and environmental cues to support adherence. Many engaged in “mental replay,” retracing their day to confirm whether medications had been taken (Gualtieri et al., 2025). Others adopted habit stacking, linking medication-taking to routine behaviors such as brushing teeth or preparing meals. Environmental cues such as visible storage locations and alarms further reinforced adherence. These strategies highlight the salience of contextual reminders, suggesting that adherence support may benefit from leveraging routines and low-burden behavioral nudges (Gualtieri and Rosenbluth, 2023).

### Interpretation through behavioral frameworks (COM-B, HBM)

Interpreting these findings through established frameworks provides deeper insight into adherence dynamics. Within the COM-B model, participants’ knowledge gaps and limited health literacy reflected capability barriers, while family reminders and environmental cues demonstrated the role of opportunity (West and Michie, 2020). Motivation emerged in participants’ desire to remain healthy for their children and in trust—or mistrust—toward clinicians. Similarly, the Health Belief Model illuminates how perceived severity of illness, perceived benefits of adherence, and barriers such as cost or cultural mistrust shaped adherence behaviors (Alyafei and Easton-Carr, 2025). Cues to action, such as alarms or family support, reinforced medication use, while self-efficacy was strengthened when participants felt capable of integrating medications into their daily lives.

### Strengths, methodological reflections, and limitations

This study’s strength lies in its exploratory design, which provided rich insights into the lived experiences of racial/ethnic minority patients managing chronic medications. At the same time, several methodological considerations must be acknowledged. The sample size was small and not representative, and the distribution across conditions and minority groups was uneven. All participants were English-speaking, another possible limitation. Recruitment relied on convenience sampling, which may have introduced bias toward individuals already engaged with health research, especially with the use of ResearchMatch as the secondary recruitment tool after posted flyers yielded one participant. While thematic saturation was observed in recurring patterns of barriers and facilitators, the small number of participants limits claims of full conceptual saturation. In addition, although the semi-structured interview guide was piloted and iteratively refined, it has not been formally validated, which may affect reproducibility. These limitations underscore that the findings are descriptive and hypothesis-generating, especially as pertains to the subgroup patterns, rather than generalizable.

### Future research directions

Future research should extend these exploratory findings by recruiting larger and more diverse samples across racial/ethnic minorities, as well as condition-specific populations. Mixed-methods approaches could help validate and build upon the patterns observed here, ensuring that future work can better assess generalizability. While this study highlights potential roles of habit stacking, environmental reminders, and family support, these observations should be viewed as preliminary and hypothesis-generating. Subsequent studies are needed to confirm whether such strategies are effective and acceptable across broader populations. By grounding future work in both theory and patient experience, researchers can gradually build the evidence base necessary to inform intervention design.

## Conclusion

The findings of this study offer exploratory insight into the home medication management decisions and practices of a small sample of a diverse cohort as well as the potential influence of race, ethnicity, family, and culture on their medication behaviors. While most participants did not report a direct link between their identities and adherence, many described how medication-taking was normalized in their households through family modeling, initial reliance on herbal remedies, with eventual adoption of pharmaceuticals following discussions with providers or personal research. Although Western medicine was generally accepted, some participants expressed a belief in minimizing its use when possible.

Although the study’s sample size was relatively small and not intended to support statistical inference, certain descriptive patterns were observed. For example, adherence scores appeared lower among participants identifying as Black/African American or Hispanic/Latino, while those identifying as Asian scored in the moderate range. These trends are not generalizable but may suggest areas for further inquiry into how cultural background and upbringing interact with adherence behaviors.

Additional findings highlight the complex and individualized decisions participants make regarding medication storage and recall strategies. Several participants described using visual cues, location-based routines, and mental review of daily activities to support adherence. Family involvement, or, in the form of providing or receiving medication reminders, was commonly reported and may be associated with improved adherence among some participants.

Overall, these qualitative insights may help inform the development of tailored interventions that consider the roles of many factors in guiding better medication adherence. The study indicates that factors to evaluate include medication storage locations, storage containers, personal routines, cultural context, and family dynamics as supports to improve medication adherence.

## Data Availability

The original contributions presented in the study are included in the article/supplementary material, further inquiries can be directed to the corresponding author.
